# Nanobody-engineered CLL-1 CAR T cells: A strategy for safer targeting in acute myeloid leukemia

**DOI:** 10.1016/j.omton.2026.201217

**Published:** 2026-05-07

**Authors:** Chakrapani Tripathi, Frank Luh, Yun Yen

**Affiliations:** 1 Sino-American Cancer Foundation, Covina, CA 91722, USA

## Main text

In our recent publication in *Cancer Research Communications*, *“*Nanobody-Engineered CLL-1 CAR T Cells: Optimizing Tumor-Specific Cytotoxicity and Minimizing Off-Tumor Toxicity*,”* we developed a next-generation nanobody-based chimeric antigen receptor (CAR) T platform targeting CLL-1 for acute myeloid leukemia (AML). Leveraging llama-derived single-domain antibodies (VHHs), we engineered highly specific CAR constructs against CLL-1 and CD33 and systematically evaluated their functional performance across *in vitro* and *in vivo* models.

CLL-1 targeted CAR T cells exhibited potent, antigen-dependent cytotoxicity against AML cells, coupled with robust T cell activation and cytokine secretion, while notably lacking tonic signaling, indicative of a well-regulated activation profile. Critically, in contrast to CD33-directed CARs, CLL-1 CAR T cells demonstrated a favorable safety profile by sparing normal hematopoietic stem and progenitor cells, as evidenced by preserved colony-forming capacity and intact multilineage differentiation potential.

In AML xenograft models, CLL-1 CAR T therapy achieved sustained tumor clearance and durable disease control, with minimal off-tumor toxicity, underscoring its translational potential as a safer and more selective immunotherapeutic strategy for AML.

CAR T cell therapy has transformed the treatment of several hematologic malignancies, particularly B-cell leukemias and lymphomas.^1^^,^^2^ However, translating CAR T cell therapy to AML remains challenging.^3^ AML is characterized by the uncontrolled proliferation of immature myeloid cells that disrupt normal hematopoiesis, leading to rapid disease progression and poor clinical outcomes.^4^ Despite the exploration of multiple targets, the lack of leukemia-specific antigens and overlap with normal hematopoietic progenitors limit both the safety and durability of CAR-based therapies.^3^^,^^5^ These limitations highlight the need for CAR designs that combine potent antitumor activity with minimal hematopoietic toxicity.

CD33 is a widely studied myeloid target,^5^ but its expression on normal hematopoietic stem and progenitor cells increases the risk of prolonged myelosuppression and off-tumor toxicity.^3^ This limitation has motivated the search for safer targets that can selectively eliminate leukemic cells. C-type lectin-like molecule-1 (CLL-1/CLEC12A) is widely expressed on AML blasts and leukemic stem cells but largely absent from normal hematopoietic stem cells,^4^ making it an attractive candidate for CAR-based therapy. Its differential expression enables precise targeting of malignant cells while sparing healthy hematopoietic compartments, addressing a major safety concern in AML immunotherapy.

Recent advances in protein engineering have further expanded the possibilities for CAR design. Nanobody (VHH)-based recognition domains derived from camelid heavy-chain antibodies offer several advantages over conventional scFvs, including smaller size, structural stability, enhanced tissue penetration, and precise antigen recognition.^6^^,^^7^ Nanobody-engineered CLL-1 CAR T cells have demonstrated robust, antigen-dependent cytotoxicity against AML cells while preserving the viability and differentiation capacity of normal progenitors.^3^^,^^8^ Functional characterization also revealed the maintenance of favorable T cell fitness, including stable proliferation, viability, and a balanced memory phenotype,^2^^,^^9^ all critical for sustained antitumor responses and long-term persistence.

Importantly, CLL-1 CAR T cells exhibit minimal toxicity toward normal hematopoietic progenitors, with preserved colony-forming potential, highlighting a key safety advantage over CD33-targeted CARs.^3^^,^^4^ Despite these promising features, challenges remain, including antigen heterogeneity and the potential for immune escape.^6^ Strategies such as dual-antigen targeting, combinatorial CARs, or logic-gated designs may enhance tumor coverage while maintaining specificity.^5^^,^^8^ Furthermore, the immunosuppressive AML microenvironment can impair CAR T cell persistence and function, suggesting that combination with immune-modulating interventions, such as checkpoint blockade or cytokine support, may be necessary to maximize therapeutic efficacy.^8^^,^^10^ Together, these insights provide a strong rationale for the continued optimization and clinical translation of nanobody-engineered CLL-1 CAR T cells in AML and related myeloid malignancies.

### Critical considerations

This study underscores key considerations for the development and clinical translation of nanobody-engineered CLL-1 CAR T cells. On-target, off-tumor toxicity remains a major concern due to low-level CLL-1 expression on normal hematopoietic cells. Antigen heterogeneity in AML raises the risk of escape and may limit long-term efficacy. *In vitro* colony assays suggest minimal progenitor toxicity, but they do not fully mimic the human bone marrow niche. Xenograft models, while informative for antitumor activity, fail to capture complete immune and cytokine dynamics. Dual-target and split CAR designs enhance specificity but increase manufacturing complexity and potential interdomain interactions. CAR T cell persistence and functional stability in patients remain uncertain. Cytokine release and immune-related adverse events require rigorous clinical evaluation. Preclinical findings provide strong translational rationale, yet cautious optimization is essential. Overall, a comprehensive assessment of safety, antigen coverage, and durability is critical for clinical success.

### Expanding scope and future strategies

**Figure 1:**
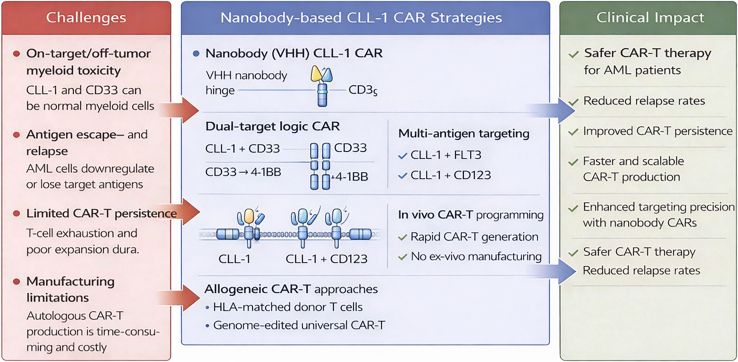
Next-generation strategies for nanobody-based CLL-1 CAR T therapy in AML

In a broader context, nanobody-based CLL-1 CAR T cells represent a highly promising and adaptable platform for precision immunotherapy in AML, combining potent and selective antitumor activity with a reduced risk of off-tumor toxicity. The intrinsic advantages of VHH-based targeting domains, including their small size, structural stability, and ability to access cryptic epitopes, enable the development of next-generation CAR designs with improved tumor specificity and functional control. Future efforts should focus on refining dual- and multi-antigen targeting strategies, such as logic-gated or split-CAR systems, to mitigate antigen escape and address the heterogeneity of AML. In parallel, advancing *in vivo* CAR T engineering approaches holds significant potential to simplify manufacturing and enhance accessibility. A comprehensive evaluation of long-term persistence, exhaustion profiles, and safety in clinically relevant settings will be critical for successful translation. Moreover, integrating these CAR T platforms with complementary modalities, such as gene editing, epigenetic modulators, or targeted therapies, may further augment their efficacy and durability. Expanding validation in patient-derived xenograft models and across diverse AML subtypes will strengthen the translational and clinical relevance of this approach, ultimately supporting the development of more precise and durable immunotherapeutic strategies (Figure 1).

### Conclusion

Nanobody-engineered CLL-1 CAR T cells represent a promising strategy to address one of the central challenges in AML immunotherapy: the identification of targets that effectively eliminate leukemic cells while preserving normal hematopoiesis. By combining the selective expression profile of CLL-1 with the structural advantages of nanobody-based antigen recognition domains, this platform offers a potentially safer and more precise CAR T approach for myeloid malignancies. Continued optimization of CAR design, evaluation in clinical trials, and integration with multi-antigen targeting or combination immunotherapies will be critical to fully realize the therapeutic potential of this strategy for patients with AML.

## Acknowledgments

This study was supported by the Sino-American Cancer Foundation Drug Discovery Research Fund and in association with the Taipei Medical University Research Center of Cancer Translational Medicine of the Higher Education Sprout Project and Taiwan Ministry of Education.

## Declaration of interests

C. T., F.L., and Y.Y. report a patent to USPTA patent file 19/259,537, pending.
